# Raman scattering and red emission of Mn^4+^ in La_0.7_Sr_0.25_Na_0.05_Mn_0.7_Ti_0.3_O_3_ manganite phosphor for LED applications

**DOI:** 10.1039/d0ra04033a

**Published:** 2020-06-22

**Authors:** Z. Raddaoui, S. El Kossi, B. Smiri, Thamraa Al-shahrani, J. Dhahri, H. Belmabrouk

**Affiliations:** Laboratoire de la Matière Condensée et des Nanosciences, Université de Monastir, Faculté des Sciences de Monastir Avenue de l'environnement 5019 Monastir Tunisia; Laboratoire de Micro-optoélectroniques et Nanostructures, Université de Monastir, Faculté des Sciences Monastir Avenue de l'environnement 5019 Monastir Tunisia; Department of Physics, College of Science, Princess Nourah Bint Abdulrahman University Riyadh Saudi Arabia; Department of Physics, College of Science at Zulfi, Majmaah University Zulfi 11932 Saudi Arabia Ha.Belmabrouk@mu.edu.sa

## Abstract

The vibrational and optical properties of an La_0.7_Sr_0.25_Na_0.05_Mn_0.7_Ti_0.3_O_3_ (LSNMT) polycrystalline sample produced *via* a solid-state reaction were studied. The Raman spectrum at room temperature reveals the chemical disorder in our compound. The optical gap and Urbach energy were estimated on the basis of the absorption spectrum. Moreover, the polycrystalline manganite radiates in the near-infrared light (1000 nm) with 514.5 nm light excitation and in the temperature range from 10 K to 300 K. Crystal field analysis suggests that only the Mn^4+^ luminescent center is found in LSNMT. The measured activation proves that our compound possesses good thermostability. The chromaticity coordinates prove that the emission of the LSNMT sample occurs in the near-infrared region. All analytical findings demonstrate that LSNMT manganite has substantial prospective applications in white luminescent devices.

## Abstract 2

The vibrational and optical properties of an La_0.7_Sr_0.25_Na_0.05_Mn_0.7_Ti_0.3_O_3_ (LSNMT) polycrystalline sample produced *via* a solid-state reaction were studied.

## Graphical abstract

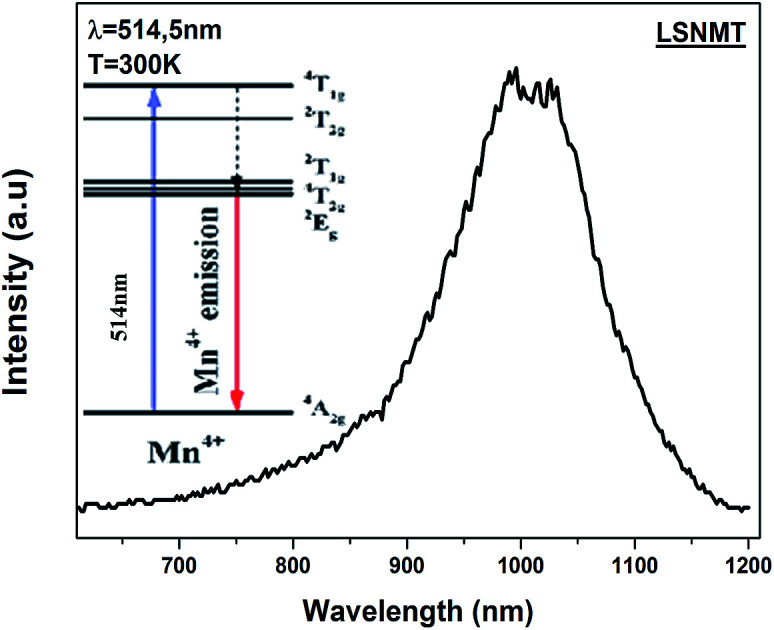

## 1. Introduction

Due to the warning from numerous research committees on pollution issues against the global industrial development, a clean nanotechnology system including multifunctional nanoparticles has become important owing to their potential applications in several research fields with actual and more efficient impacts in several areas of research.^1–3^

More precisely, manganite-based perovskite-type oxides of the composition A_1−*x*_B_*x*_MnO_3_ have appeared to be an attractive class of performing materials because of their low cost and abundance on the earth.^4,5^ Their diversity and promising physical properties deserve significant interest in numerous nanotechnology-related applications such as optoelectronic devices, electrocatalysis, and memory storage devices. For these reasons, worldwide research activity is devoted to understanding the origin of these potential characters.^6,7^

However, the functionality of manganite is well known for its magnetic properties and particularly colossal magnetoresistive behavior. These features represent the most important discovery and revolution of the magnetic system. It is accompanied by the magnetic transition, which is explained by the double exchange and superexchange phenomena.^8^ Usually, magneto-transport characteristics have been connected to the double exchange phenomena, which are governed by the movement of the e_g_ electrons from Mn^3+^ to Mn^4+^.^9^ These proprieties have attracted considerable attention and bring a new theory and model to explain the interactions between transport and magnetic behaviors such as spin-glass and charge ordering.^10^

The simplicity of structure has been extensively exploited as a clue for numerous applications due to the moderation of properties by the substitution of A and/or B, resulting in a variety of structures that could play a key role to manipulate capacity and performance.^11^ The occupation of the transition metal B in the octahedral system produced a splitting of the d orbital into two degenerated e_g_ orbitals and three t_2g_ orbitals. This step was pursued following the effect of Jahn–Teller to create a stable structure with two energies states (σ*, σ) in e_g_ orbital occupation and the covalent oxygen-related intermediate species.^12^ In fact, the variation of the ionic radii is a type of chemical pressure that creates a deformation in BO_6_ from the perfect ceramic, which causes large degrees of strain and distorts the surrounding lattice and yields a variety of unit cells and lattice parameters.^13^ Full knowledge of these factors is important to underline the high impact of microstructural properties on the double exchange phenomena that is the source of the physics of the properties in perovskites. This strongly relies on the B–O bond length (*d*_B–O_) and the (*θ*_B–O–B_) bond angle.^14^

Strontium-substituted lanthanum manganite (La_0.7_Sr_0.3_MnO_3_) as a typical magnetic material opens the door to numerous investigations on the origin of its colossal magnetoresistive properties. These properties are explained in terms of the interaction between the structural distortion and magnetic mechanisms related to the magnetic coupling between Mn^4+^ and Mn^3+^. Abdelmoula *et al.*^15^ reported that the substitution by a monovalent element can improve the physical properties of manganite. In fact, they investigated the impact of Na incorporation in La_0.7_Sr_0.3_MnO_3_, which proves that the doped manganite oxides (La_0.7_Sr_3−*x*_Na_*x*_MnO_3_) constitute very attractive candidates as potential giant magneto-resistance compounds. Similarly, Kossi *et al.*^16^ have reported that La_0.7_Sr_0.025_Na_0.05_MnO_3_ and La_0.7_Sr_0.025_Ka_0.05_MnO_3_ show a giant magnetocaloric property and announced giant dielectric properties with the partial substitution of Mn by Ti. In the same way, more recent studies proved that La_0.7_Sr_0.3_MnO_3_ and its derivatives can be the next-generation energy technologies for clean power generation due to their multiferroic properties and their hopeful applications in numerous domains.^17^

Moreover, recent progress reveals that manganites present alternative materials to phosphorus as a luminescent matrix and open up new fields of investigation.^18^ However, much work remains to be done to achieve high luminescence performance adaptable to large-scale technological applications. In fact, manganite is influenced by the doping ion, *i.e.*, manganese ion (Mn^4+^). This tetravalent ion is luminescent. It is able to emit red light when it is excited by blue or near-UV light.^19^ In addition, the luminescence characteristics can be tailored and enhanced by varying the dopant concentration, grain size, synthesis process, and temperature of sintering. However, the most important factor influencing these characteristics is the choice of the dopant and the doping site that directly affect the characteristic of MnO_6_ octahedrons.^20^

In this study, LSNMT polycrystalline manganite was prepared *via* a classical solid-state method that requires high temperatures. The vibrational properties of the LSNMT manganite were studied *via* Raman spectroscopy. The luminescence characteristics of our manganite compound were investigated from its absorption spectrum and photoluminescence (PL) spectrum. The thermal stability of LSNMT was deduced from its PL spectra, which depends on the temperature value.

## 2. Background

Raman spectroscopy is a well-known technique that provides the chemical and structural knowledge of diverse compounds using the data of the vibrational states of a matter. Raman-shifted photons have either Stokes scattering or anti-Stokes scattering. Since phonons are bosons, the Stokes and anti-Stokes intensities are respectively related to (*n* + 1) or *n*. The quantity *n* denotes the occupation number. Since the Stokes intensity is more intense than the anti-Stokes intensity, only the Stokes intensity is usually registered and interpreted in traditional Raman spectroscopy.^21^

In general, to compute the intensity of the compounds during Raman scattering some equations are required. We recall hereafter some of these equations. Let *ℏ* be the reduced Planck constant, *ω*_i_ denotes the angular frequency of the incident photon, *ω*_s_ is the angular frequency of the scattered photon, *ω*_p_ is the angular frequency of the scattered phonon, *k*_B_ is the Boltzmann constant and *T* is the absolute temperature.

It is well known that the Stokes process corresponds to the emission of a phonon, whereas the anti-Stokes process corresponds to the absorption of a phonon. We deduce that the energy change during these phenomena is given by:1
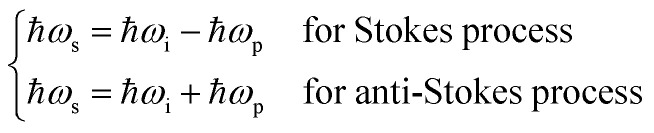


The intensities *I*_S_ and *I*_AS_ of the first-order scattering by a phonon or more generally by a boson are given by:2
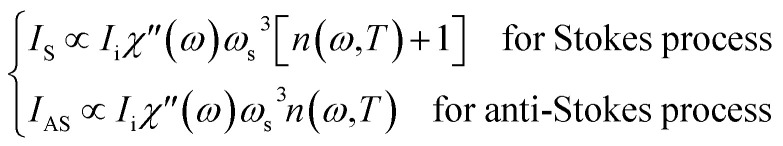
where *χ*′′(*ω*) is the line shape response of a harmonic mode.

The occupation number *n*(*ω*,*T*) of phonons at thermal equilibrium is given by:3
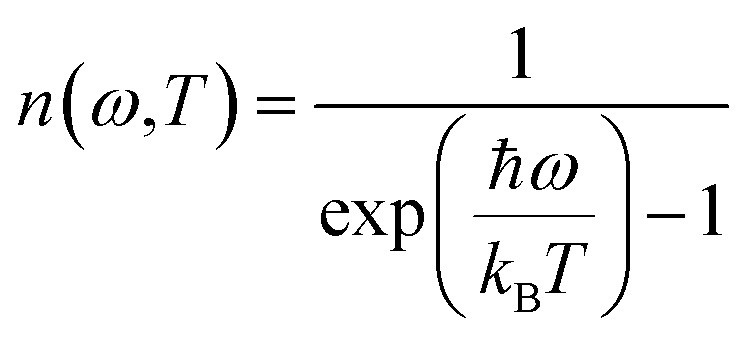


The ratio of the intensities related to the second-order scattering is approximated by the following equation:4
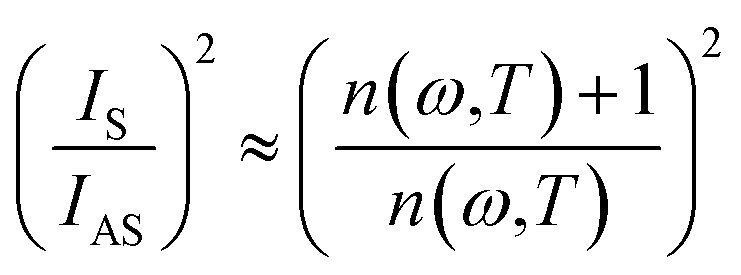


Optical characteristics are one of the most interesting parameters to estimate the light efficiency. One of them, the optical band gap (*E*_g_) can be obtained by employing the basic absorption.

They are determined using the following formula:^22^

5 *αhν* = *B*(*hν* − *E*_g_)^*n*^

A crystallized ceramic is ideally perfect. The absence of this condition is attributed to a defect in the solid, which can be connected to a defect caused by impurities and distortion.

The Urbach energy *E*_u_ describes the disorder of a sample.

From the change in the absorption coefficient, it is possible to extract the disorder in the sample, resulting from this equation.

6 *α* = 2.303 × (*A*/*d*)

7
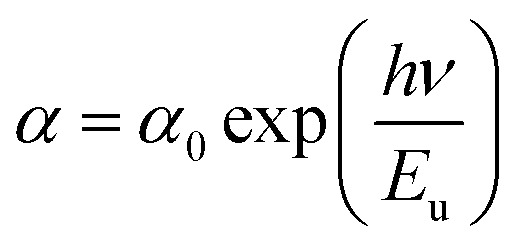
where *d* is the thickness and *A* is the optical absorbance of the sample.

## 3. Experimental procedure

**Fig. 1:**
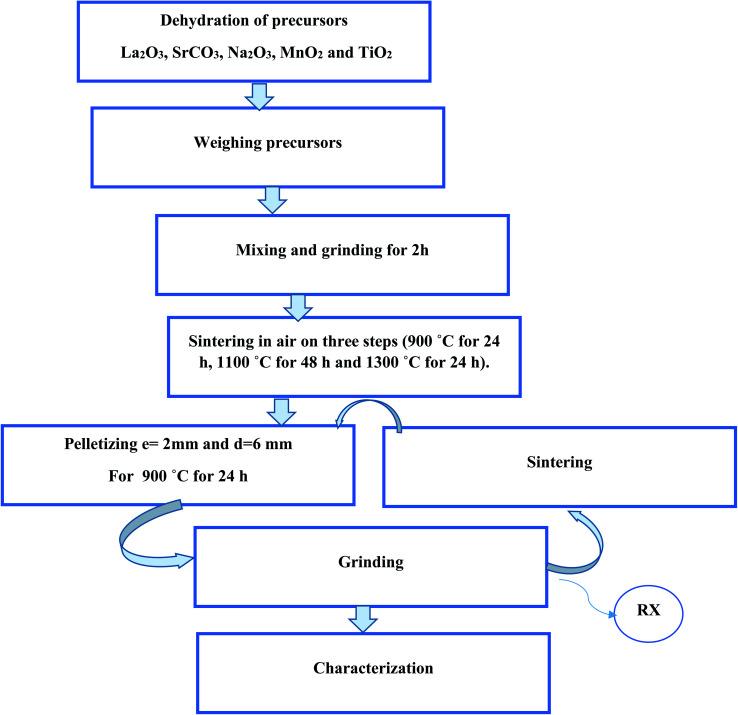
Flow-chart of LSNMT prepared by a solid-state method.

The LSNMT sample was produced by the well-known solid-state method. The metal precursors La_2_O_3_, SrCO_3_, Na_2_O_3_, MnO_2_ and TiO_2_ were of high purity (99%). The polycrystalline manganite preparation has been discussed in our previous work.^16^ Furthermore, Fig. 1 depicts the steps of the process of synthesis.

The X-ray structure analysis by the Rietveld refinement was discussed in our previous work.^16^ It was noted that LSNMT crystallizes in the rhombohedral *R*3̄*c* structure without any detectable secondary phase, with lattice parameters *a* = *b* = 5.536(3) Å, *c* = 13.438(3) Å and cell volume *V* = 356.75(2) Å^3^. It is also important to recall the values of the bond length *d*_Mn–O–Mn_ = 1.97(1) Å and the bond angle *θ*_Mn–O_ = 164.24(3)°.

The Raman data were recorded in the frequency range 80 to 1000 cm^−1^ using a LABRAM HR800 Raman spectrometer. The spectral resolution of the system was 3 cm^−1^ with a power of about *P* = 50 mW and ×10 objective (focus diameter larger than 10 microns). The scattered radiation was detected by a 1024 × 256 CCD camera. Our compound was excited by a 532 nm laser at room temperature.

The UV-Vis-NIR spectra at room temperature in the wavelength range of 150 to 1500 nm were measured on a Shimadzu UV-3101PC spectrophotometer with a source emitting wavelength radiations on a pellet of our compound.

The photoluminescence (PL) data were recorded between 10 K and 300 K while keeping the sample in a closed-cycle helium circulation cryostat. The sample was excited by employing a 514.5 nm line of the continuous wave Ar^+^ laser and a situated power excitation of 50 W cm^−2^. Spectral analysis of the luminescence measurements was dispersed using Jobin Yvon HRD1 monochromator and detected by a thermoelectrically-cooled Si photodetector.

## 4. Results and discussion

### 4.1. Raman scattering

**Fig. 2:**
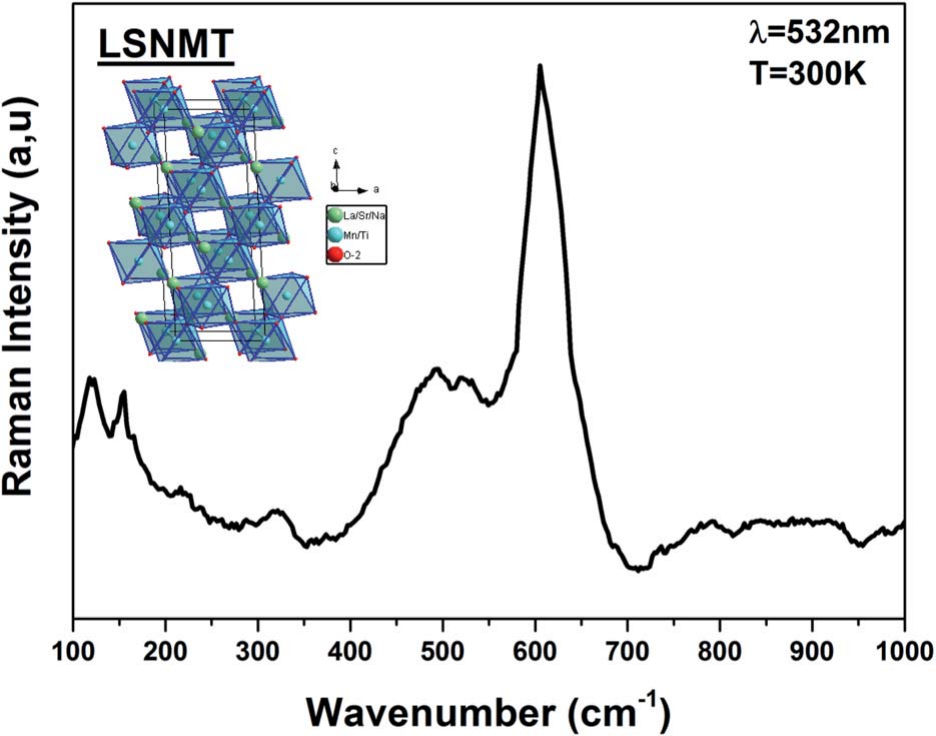
Raman spectrum of the LSNMT ceramic at room temperature for excitation 532 nm. Inset the crystal structure and the TiO_6_ octahedron for our compound.

Recently, several papers argued that optical properties in manganite depend on the Mn–O bond length and the symmetry of MnO_6_ confirming the correlation of optical phonon mode with the degree of activation of the Jahn–Teller distortion modes.^23^ Usually, Jahn–Teller effects have a major role in the rhombohedral structure manganite related to six equal Mn–O bond lengths governing the dynamic and non-coherent deformation of the MnO_6_. Nevertheless, it is well known for this distorted structure that just five Raman-active modes can be detected linked to vibration and stretching oxygen vibrations of the MnO_6_.^24,25^ For more clarification on the importance of the structural properties and their effect in the optical results, the ABO_3_ type perovskite structure with (La/Sr/Na) and (Mn/Ti) cations at A-site and B-site, respectively, is illustrated using “Diamond” program (inset Fig. 2).

After dividing by the factor *n*(*ω*) + 1, the Raman spectrum of rhombohedral LSNMT manganite in the frequency [80–1000 cm^−1^] is illustrated in Fig. 2. The obtained Raman spectrum of LSNMT ceramic exhibits similar behavior in position and profile to that of the data from LaMnO_3_, which is characteristic by the rhombohedral phase (*R*3̄*c*).^26–28^ In our case, we observed two peaks in the intervals of 100–300 cm^−1^ and 400–700 cm^−1^ associated with phonons of the rhombohedral phase and can be centered at five Raman modes 117, 156, 493, 518 and 605 cm^−1^. The band at lower frequencies occurs around 117 cm^−1^ from designing the hardening of A_1g_ phonon mode related to the dominant A-site cations (La/Sr/Na) distortions and the electron–phonon coupling strength.^29,30^ Also, the peaks at 156 and 493 cm^−1^ are assigned as *E*_g_ symmetry mode identified as an internal mode (bending of the MnO_6_ octahedra) and we ascribe the two highest peaks at 605 and 518 cm^−1^ to *E*_g_ bands defined by the vibration of oxygen in MnO_6_ octahedra.^31,32^ The proposed assignments and other observed mode wavenumbers are summarized in Table 1, which compares the patterns observed in this study with those recorded according to the several previous research works such as La_0.67_Ba_0.25_Ca_0.08_Mn_(1−*x*)_Ti_*x*_O_3_ by M. Bourguiba *et al.*^33^ and La_0.65_Eu_0.05_Sr_0.3−*x*_MnO_3_ by R. Bellouz *et al.*^34^

**Table 1:** The mode vibration of the Raman spectra of LSNMT at room temperature

Raman modes	La_0.7_Sr_0.25_Na_0.05_Mn_0.7_Ti_0.3_O_3_ our compound	La_0.67_Ba_0.25_Ca_0.08_Mn_(1−*x*)_Ti_*x*_O_3_ for *x* = 0.00 (ref. 33)	La_0.65_Eu_0.05_Sr_0.3−*x*_MnO_3_ for *x* = 0.00 (ref. 34)
	Frequency (cm^−1^)	Frequency (cm^−1^)	Frequency (cm^−1^)
A_1g_	117	110	—
E_g_	156	180	—
A_1g_	—	—	310
E_g_	—	—	369
E_g_	493	490	495
E_g_	518	—	—
E_g_	605	605	650

However, the Raman scattering confirms that the crystal lattice and symmetry distortion affect the Mn^4+^ environment and the optical properties in our compound. The same result was reported for other samples such as La_0.67_Ca_0.33_Mn_1−*x*_V_*x*_O_3_.^35,36^

### 4.2. Absorption spectrum

**Fig. 3:**
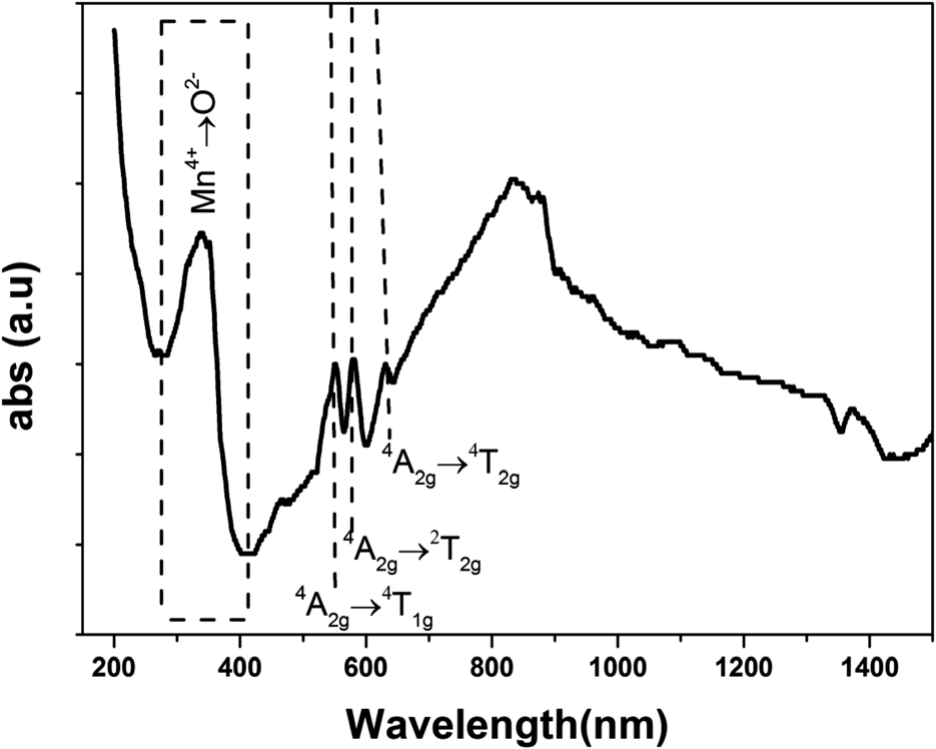
Absorption spectrum of the LSNMT ceramic in the UV-Vis-NIR region at room temperature.

The UV-Vis-NIR absorption spectrum of LSNMT ceramic at room temperature is displayed in Fig. 3. The attribution of the noted absorption bands has been made based on previous research on tetravalent manganese ion (Mn^4+^) doped perovskite ceramics.^37^ The absorption band from 200 nm to 400 nm is related to the charge-transfer (CT) transition from Mn^4+^ to O^2^ while three strong absorption peaks in the band from 400 to 700 nm are the contributions of ^4^A_2g_ → ^2^T_2g_, ^4^A_2g_ → ^4^T_1g_, and ^4^A_2g_ → ^4^T_2g_ spin-allowed d–d transitions, respectively, of Mn^4+^ ions.^38^ Also, we can see an intense asymmetric wide far-red band referred to a significant overlap of six distinct anti-Stokes and Stokes sidebands between 650 and 900 nm related to the various vibrational modes of 2E_g_ → 4A_2g_ transitions in the [MnO_6_]^8−^ octahedra for the 3d^3^ electrons.^39^ The high absorption efficiency mentioned that Mn^4+^ ion has considerable effects on the comportment of the LSNMT to emit red light.

**Fig. 4:**
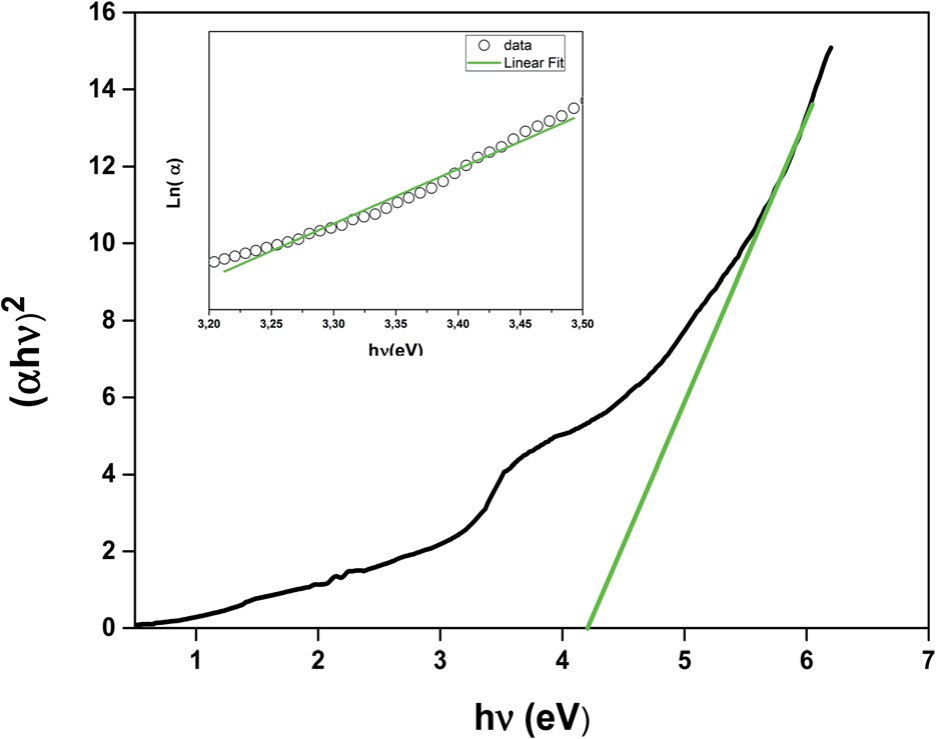
Energy band gap as a function of the LSNMT ceramic. Inset the plot ln(*α*) *versus* photon energy (*hν*) for LSNMT.

We studied the light absorption for our compound; in our sample, the absorbance spectrum was procured and presented in Fig. 4. The optical gap of our sample could be estimated by the following equation:^22^

8 (*αhν*)^2^ = *B*(*hν* − *E*_g_)

where *α*, *hν*, and *B* are the absorption coefficient, the energy of the photon, and a constant, respectively.

To compute the energy *E*_g_ of the LSNMT sample, we plot (*αhν*)^2^*vs.* (*hν*). We obtain *E*_g_ = 4.1 eV for the La_0.7_Sr_0.25_Na_0.05_Mn_0.7_Ti_0.3_O_3_ compound. This value is similar to that obtained for other perovskites as reported by Kumar *et al.*^39^

In addition, the electronic bandwidth (*W*) could be determined by the following equation:^40,41^*W* ∝ cos[1/2(π − (Mn/Ti–O–Mn/Ti))]/*d*_Mn/Ti–O_^3.5^where (Mn/Ti–O–Mn/Ti) is the bond angle and *d*_Mn/Ti–O_ is the bond length. We obtain the value *W* = 9.23 × 10^−2^ a.u.

In addition, the energy *E*_g_ is related to *W* by the equation; *E*_g_ = *Δ* − *W*, where *Δ* is the energy of charge-transfer.^42^ The width of defect bands produced in the bandgap is related to *E*_u_.^43^ It should be calculated from the formula eq:9
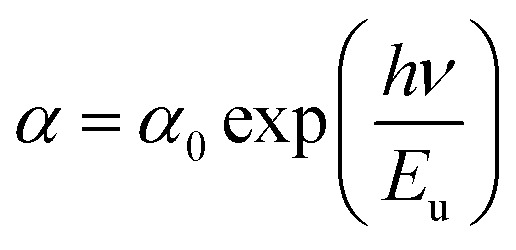


The Urbach energy *E*_u_ was measured by tracing ln(*α*) *vs. hν* (inset in Fig. 4). The value Urbach energy *E*_u_ obtained in the present work are found to be in low 0.227 eV for the LSNMT ceramic. This suggests the same behavior as reported in other research works, such as on Mn^4+^-doped glasses.^44,45^

### 4.3. Photoluminescence properties

**Fig. 5:**
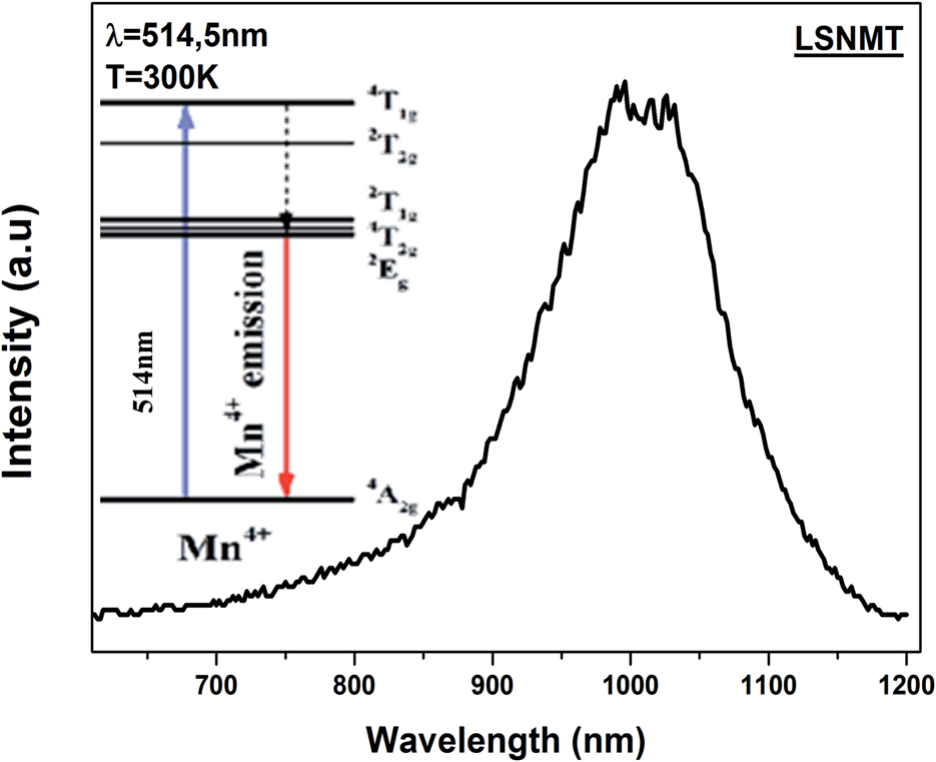
The emission spectrum of the LSNMT ceramic excited at 514.5 nm at room temperature. Inset schematic energy level scheme of Mn^4+^ ions.

The room temperature emission spectrum of the LSNMT ceramic in the Vis-NIR region is illustrated in Fig. 5. Here in our case, we can observe strong emission peaks below 1000 nm. These peaks originate from the charge transfer transition of Mn^4+^–O^2−^ and spin-allowed transitions of Mn^4+^: ^4^A_2g_ → ^4^T_1g_, ^4^A_2g_ → ^4^T_2g_ (see in inset Fig. 5), the same behavior as reported previously by X. Gao *et al.*^45^

The working temperature of polycrystalline LSNMT manganite will be lower than the room temperature.

**Fig. 6:**
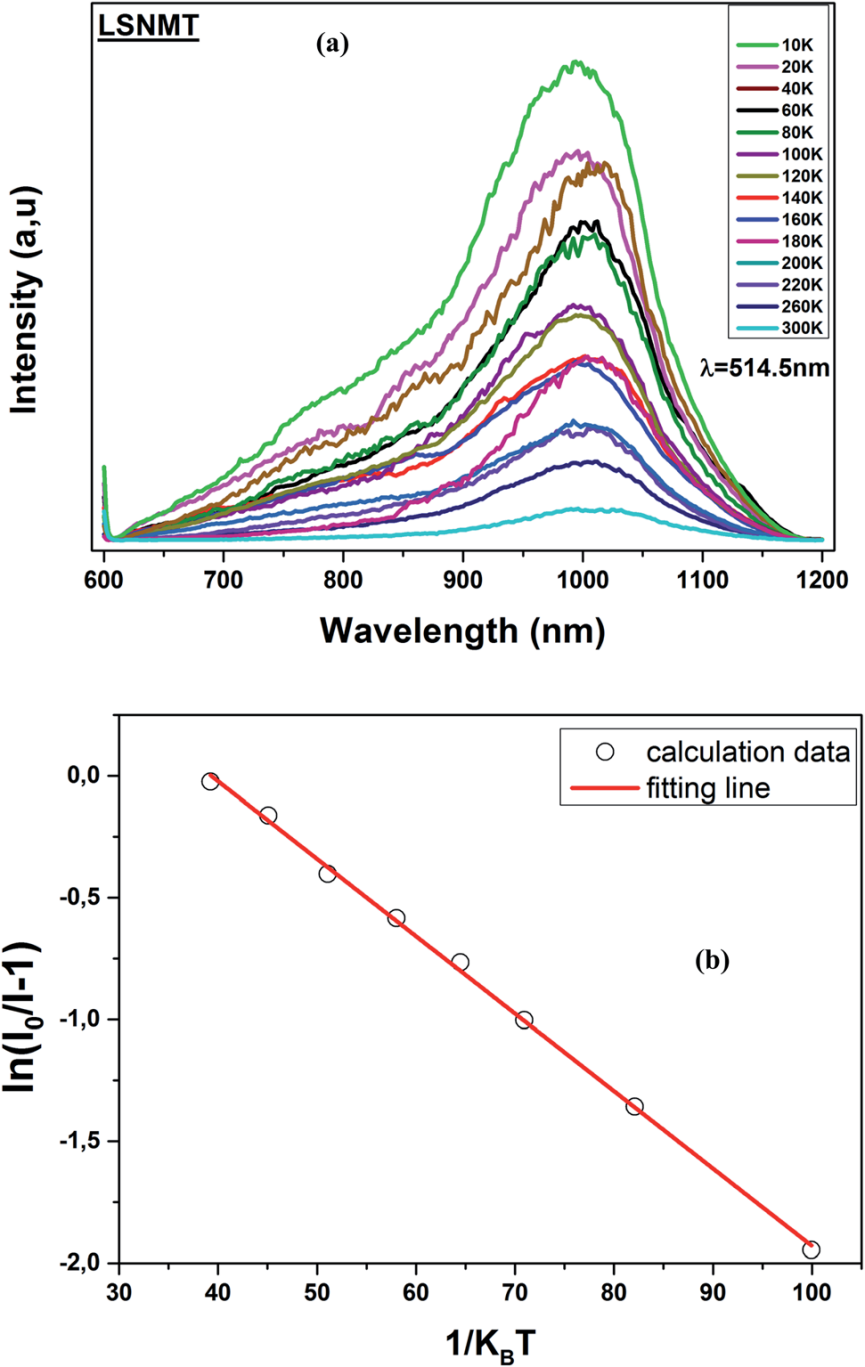
(a) Temperature-dependent PL emission spectra of the LSNMT ceramic, (b) ln[(*I*_0_/*I*) − 1] and 1/*k*_B_*T* for LSNMT.

Consequently, thermal stability is particularly important in a manganite. Fig. 6(a) displays the temperature-dependent PL spectrum (*λ*_ex_ = 514.5 nm) over the temperature range of 10–300 K for the LSNMT manganite. We notice that the PL intensity decreases with rising temperature due to nonradiative electron transitions increasing with rising temperature.^46,47^

The activation energy (*E*_a_) was calculated by Arrhenius equation as given in:^48^10
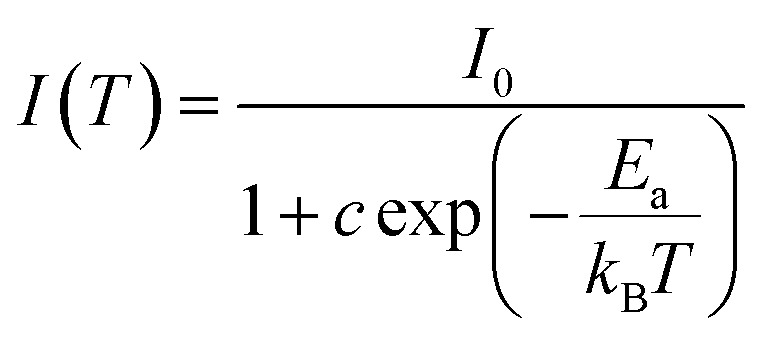


This equation can be transformed into the following form:11
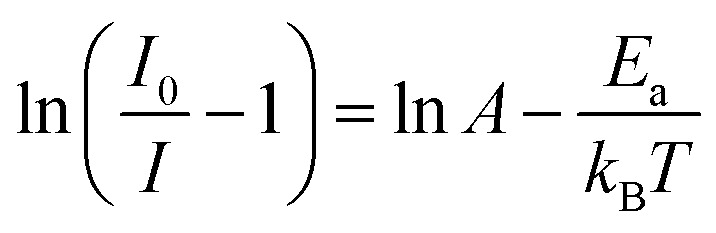
where *I*_0_ is the PL intensity at the 10 K, *A* is a constant and *k*_B_ is the Boltzmann constant (8.629 × 10^−5^ eV K^−1^).

The *E*_a_ (0.318 eV) was obtained by plotting ln[(*I*_0_/*I*) − 1] and 1/*k*_B_*T* as seen from the plot in Fig. 6(b). This result suggests that LSNMT manganite ceramic present good thermal stability.

**Fig. 7:**
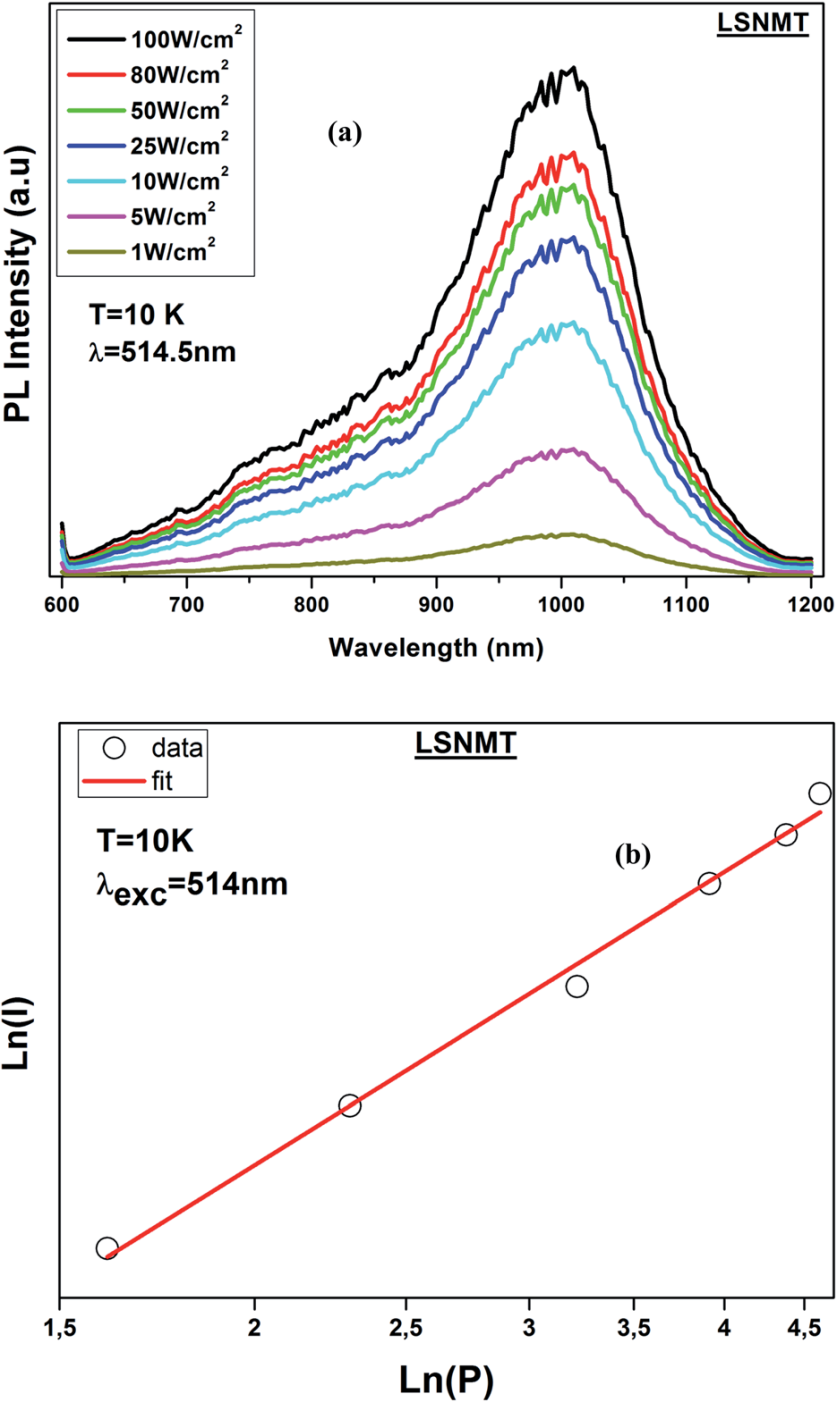
(a) PL intensity of LSNMT at 10 K at different excitation power densities, (b) log–log plot of PL intensity *versus* excitation power density for ceramic at 10 K.

In addition, the PL intensities spectra of the LSNMT ceramic using a 514.5 nm laser excitation for the selected excitation power at 10 K, are displayed in Fig. 7(a). It should be noted that the PL intensities rise with an increase of the excitation power and the position of the emission spectra remained constant as the excitation power is raised.

The correlation between PL intensity (*I*) and the excitation power (*P*) can be described by the following equation:^48^

12 *I* ∝ (*P*)^*n*^

where *n* is the number of photons. Fig. 7(b) shown the linear plot of ln(*I*) *vs.* ln(*P*) as shown in Fig. 7(b) for the band emission for Mn^4+^. The fitted *n* values for the band emission are 2, suggesting that a two-photon process has helped to populate the transition in this sample.

**Fig. 8:**
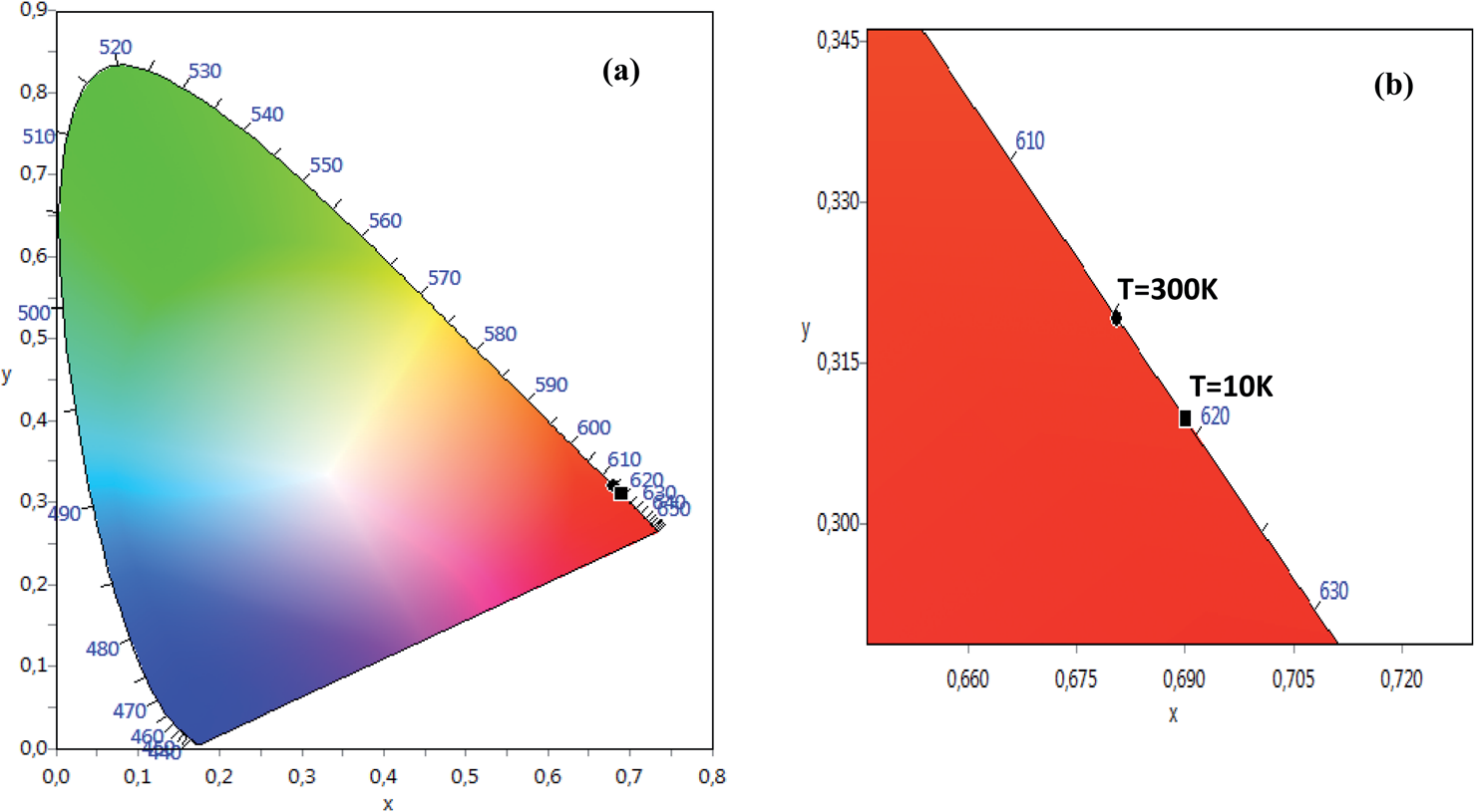
(a) The CIE chromaticity of the LSNMT sample at room and at low temperature (10 K), (b) the grow of the CIE chromaticity.

The CIE chromaticity of the LSNMT sample at room and at low temperature (10 K) are represented in Fig. 8. The CIE diagram exhibit that the approximate coordinates were related in the red region. The values of CIE coordinates of the LSNMT ceramic are tabulated in Table 2. The resulting CIE chromaticity coordinates of our compound at room and at low temperature (10 K) are (0.6806, 0.3192), (0.6901, 0.3097), respectively.

**Table 2:** The values of CIE (*x*, *y*) co-ordinates of LSNMT at room temperature and 10 K

La_0.7_Sr_0.25_Na_0.05_Mn_0.7_Ti_0.3_O_3_	CIE	
	*x*	*y*
At *T* = 300 K	0.6806	0.3192
At *T* = 10 K	0.6901	0.3097

New technologies based on red phosphor have attracted the attention in industrial applications as white-light-emitting diodes (W-LEDs) to avoid the high color temperature and low color index problem.

Against the high price of this compound, further research and development confirm that transition-metal Mn^4+^-doped luminescent compounds can be an alternative system to emits red light when excited by near-UV or blue light. However, our investigations of the vibrational and optical behaviors of the LSNMT has demonstrated for the first time that pure red emission persists in our crystal structure. This work can solve the problem of many applications leading to a stable structure of manganite which is correlated to the red pure emission and providing a good candidate for white luminescence display devices.

## 5. Conclusion

In conclusion, LSNMT ceramics with the polycrystalline perovskite structure have been produced by a solid-state method. The vibrational and optical behaviors were studied. Raman spectrum at room temperature confirmed the rhombohedral phase (*R*3̄*c*).

In addition, the optical gap and Urbach energy values were determined using the absorption spectrum at room temperature. The PL intensity at room temperature response emits red light (1000 nm). Temperature-dependent PL revealed that our compound has good thermal stability. Using the chromaticity coordinates (CIE), one can see that the LSNMT sample show emission in the red region. All analytical results demonstrate that LSNMT manganite has the potential for considerable applications in white luminescence devices.

## Conflicts of interest

There are no conflicts to declare.

## Supplementary Material
